# Impacts of external factors on Ethiopia's economic growth: Insights on foreign direct investment, remittances, exchange rates, and imports

**DOI:** 10.1016/j.heliyon.2023.e22847

**Published:** 2023-11-28

**Authors:** Dereje Fedasa Hordofa

**Affiliations:** Dire Dawa University, Lecturer in Economics Department, Dire Dawa, Ethiopia

**Keywords:** Ethiopia, Time series analysis, Autoregressive distributed lags modeling, External determinants, Macroeconomic policy

## Abstract

This study conducts a rigorous examination of relationships that are often assumed but rarely tested within the context of Ethiopia's journey towards sustainable development. An autoregressive distributed lag model is employed using annual time series data from 1982 to 2021 to investigate the short- and long-term impacts of foreign direct investment (FDI), remittances, real exchange rates, and imports on Ethiopia's economic growth. By incorporating additional control variables, this study contributes nuanced insights that were previously lacking in the literature. The findings reveal intriguing patterns that both align with and deviate from existing frameworks. Contrary to some prior studies, foreign investment consistently emerges as a significant driver of economic growth over time. However, remittances demonstrate only transient significance, highlighting the need for cautious policy considerations. The influence of exchange rates on economic growth proves to be unexpectedly complex and nonlinear, challenging conventional assumptions. The empirical validation of these multifaceted realities underscores the importance of this analysis. Furthermore, robustness tests conducted in this study confirm the reliability of the findings while shedding light on additional intricacies. For instance, the relationship between imports and growth is context-dependent and exhibits ambiguities that call for careful consideration. The theoretical and practical implications derived from this research offer valuable insights for researchers and policymakers alike. The recommendations put forth emphasize the promotion of sustained prosperity through evidence-based strategies that prioritize community development.

## Graphical abstract

**Figure:**
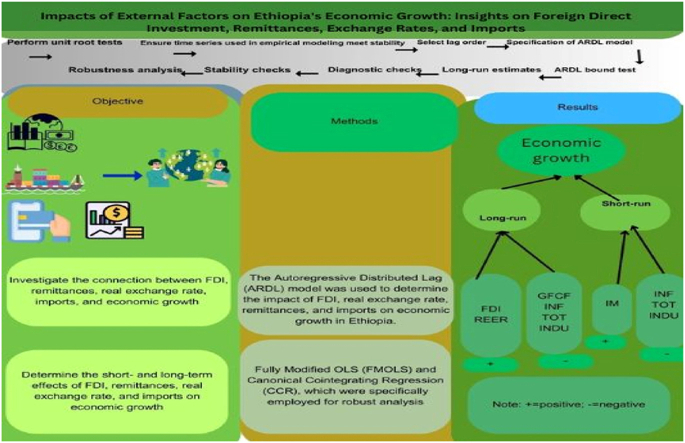

## 1 Introduction

The imperative for policymakers and scholars lies in the realization of Ethiopia's economic growth potential. In order to accomplish this objective, it is imperative to thoroughly examine the complex dynamics and significant variables that contribute to the formation of the nation's economic environment. The objective of this study is to examine the impact of foreign direct investment (FDI), remittances, the real exchange rate, and imports on the economic development of Ethiopia. Through a comprehensive analysis of the interconnections and impacts of these factors, significant knowledge may be acquired regarding the methods by which they facilitate the realization of Ethiopia's economic expansion. The role of foreign direct investment (FDI) in fostering economic growth has been well recognized. According to Ref. [1], foreign direct investment (FDI) inflows have the capacity to encourage local investment, enable the transfer of knowledge, and generate employment possibilities. In the same vein, it has been acknowledged that remittances, which refer to the monetary transactions initiated by migrants to support their countries of origin, hold significant importance as a vital revenue stream and a driving force behind economic progress [2]. Remittance flows have the potential to make substantial contributions to investment, consumption, and poverty alleviation, thereby exerting a notable influence on national economies.

Moreover, the impact of the real exchange rate on economic growth is a topic of considerable importance. A competitive real exchange rate can enhance export competitiveness, attract FDI, and stimulate economic growth [3]. On the other hand, imports play a dual role in the economy, as they provide access to goods and service that fuel domestic production while also influencing the balance of trade and affecting the overall economic performance [1]. Understanding the interplay between foreign direct investment, remittances, the real exchange rate, and imports is crucial for policymakers and researchers seeking to unlock Ethiopia's economic growth potential. By comprehensively examining the relationships and effects of these variables, this study aims to contribute valuable insights that can inform evidence-based policy decisions and propel Ethiopia towards sustainable and inclusive economic development.

The COVID-19 pandemic has significantly impacted economies globally, including Ethiopia. Studies have explored the impact of the pandemic on FDI in developing countries, such as Ethiopia [4,5]. provide insights into changes in investment patterns, investor sentiment, and policies during and after the pandemic. The relationship between the real exchange rate and economic performance during and after the pandemic is another crucial topic. Ref. [6] investigate the effects of currency devaluation on Ethiopia's major export commodities, providing valuable insights into coffee and khat. Innovation, social media, and internet use have emerged as key factors in shaping economic growth. Also [7], explore the effect of transport infrastructure and technological innovation on economic growth, energy consumption, and CO2 emissions. ref. [8] examine the economic impact of transport infrastructure in Ethiopia and the role of FDI while [9] highlight the nexus between urbanization, technological innovation, and environmental sustainability in Ethiopia and Egypt.

Ethiopia harbors untapped potential and promising opportunities within the dynamic global economy. A comprehensive understanding of the intricate factors driving its economic growth is essential in light of its increasing significance [10,11]. The existing literature on sustainable development highlights key concepts such as healthcare supply chains [12], crisis production plans [13], and environmental impacts [14,15]. These studies shed light on how renewable energy, human capital, and trade influence the environment, providing policymakers with valuable insights [14,16,17]. This extensive study also encompasses urbanization and industrialization and their implications for carbon emissions, laying the foundation for a more sustainable future [18,19]. In this complex landscape, this research becomes crucial for understanding Ethiopia's economic trajectory. Each study compiles a range of facts, showcasing Ethiopia's progress towards sustainable and resilient economic growth. Like many sub-Saharan African countries and emerging economies, Ethiopia aspires to achieve long-term economic growth. However, the forces of globalization and anti-globalization sentiments in specific sectors pose challenges in identifying the drivers of economic growth [2,20].

**Fig. 1:**
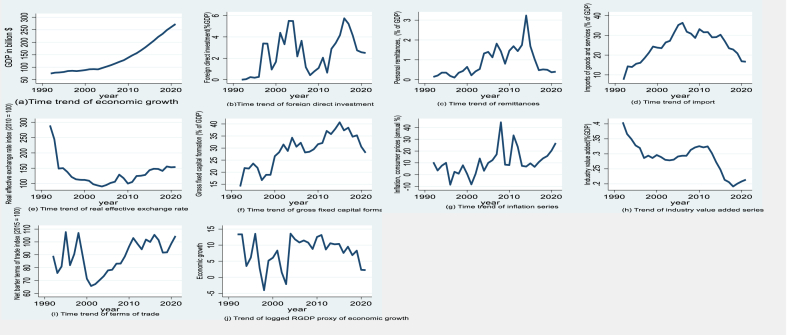
Time trends of the main study variables (1982–2021) (a) economic growth; (b) foreign direct investment; (c) remittances; (d) imports. (e) Real effective exchange rate, (f) gross fixed capital formation, (g) inflation, (h) industry value added, (i) terms of trade, and (j) log of real GDP series.

Source: Authors computation, 2023.

A deeper examination is necessary to comprehend Ethiopia's development trajectory in recent decades (Fig. 1). Persistent challenges include overreliance on agriculture, infrastructure concerns, and an unstable economy [21,22]. The COVID-19 pandemic has further impacted Ethiopia's economy by disrupting supply chains and reducing exports [21]. Exploring Ethiopia's potential for economic development becomes crucial. In a globalized world, understanding the underlying variables driving economic growth becomes increasingly complex, particularly for emerging countries like Ethiopia. Research suggests that effective macroeconomic policies, resilient institutional frameworks, stable political conditions, and national savings play a role in economic growth [21,[23], [24], [25]].

External factors have also played a significant role in boosting the economies of small, growing states. Foreign investment, remittances, imports, and currency swaps contribute to economic development [26,27]. The growing Ethiopian diaspora sends a substantial amount of money back home, with remittances steadily increasing over the years, reaching $5.8 billion in 2021. This underscores the importance of remittances to the Ethiopian economy [22]. Remittances have the potential to alleviate poverty by providing financial resources to many individuals. However, governments must carefully consider potential drawbacks, such as labor force participation and hours worked. Ensuring effective distribution of remittances to maximize benefits while minimizing any potential economic harm necessitates vigilance [28].

Ethiopia needs to analyze its different economic factors to maximize its development potential. Fig. 1 shows key factors illustrating Ethiopia's economic development. Ethiopia's GDP has grown significantly, suggesting economic success (Fig. 1, panel a). Ethiopia relies on foreign investment for economic progress. Fig. 1, panel b, highlights investment inflows' large volume and importance in economic growth. Fig. 1 panel c shows how personal remittances affect the Ethiopian economy, relieving poverty and providing income for many families. In Fig. 1, panel d, the percentage of imports to GDP shows how dependent a country is on imports and how this affects its economy. Import policy management is crucial to balancing import benefits with local industry protection. Policymakers may identify key intervention areas and create economic development strategies by analyzing these factors [20]. Maximizing Ethiopia's economic benefits requires creating a favorable investment climate, advocating for prudent macroeconomic policies, efficiently harnessing remittances and foreign direct investment, and managing import regulations. The real exchange rate, capital creation, inflation, industrial value added, and net barter terms of trade must be considered to fully comprehend Ethiopia's economic progress. The variables provide important information on macroeconomic conditions, investment patterns, price stability, industry contributions, and export-import dynamics (Fig. 1, panels e–j).

Through a detailed examination of these indicators and their implications, experts and decision-makers may understand Ethiopia's economic success and develop specific strategies to maximize its potential. Ethiopia can achieve economic success and improve quality of life by focusing on sustainable development and resource efficiency. In failed nations, sustainable growth and development may boost jobs, reduce poverty, and improve living conditions. Thus, all economic initiatives now aim for economic development and social well-being [23,26,27,29].

Previous studies have shown that foreign direct investment (FDI) has a major influence on Ethiopia's economy. Multiple studies show that foreign investment boosts Ethiopia's economy, especially in agriculture and hotels [23,[30], [31], [32]]. A favorable investment environment to attract FDI and boost economic development is crucial [[31], [32], [33]]. Other experts have noted FDI's potential influence on Ethiopia's economy [32]. Additionally, the research shows that FDI boosts Ethiopia's economy by improving transport infrastructure. Positive business policies in East Africa also attract FDI [8,32]. Studies have also explored how foreign direct investment (FDI) affects economic development in Sub-Saharan Africa, particularly Ethiopia. Scholars have shown that FDI boosts economic development in these nations, underscoring the need to promote it [34,35].

Many countries appreciate the relevance of import policy for growth. Many scholarly studies in many countries suggest that imports increase economic growth. These studies also suggest governments should be careful when establishing import controls to protect local businesses [[36], [37], [38]]. By ensuring the supply of needed products and services, import regulations have benefited Ethiopia's economy throughout the COVID-19 epidemic. Sustainable economic growth requires GDP diversification. This study adopts Dutch disease theory [39,40]. Management failures may impair commerce and economics in countries with high capital inflows [41].

Foreign direct investment (FDI), remittances, real exchange rate changes, and imports may produce Dutch disease impacts on Ethiopia's economy [41]. Different ideas imply that expanding economies may have different effects [42]. Assessing the immediate effects requires further research. Previous studies have demonstrated that FDI greatly influences Ethiopia's economic growth. Multiple studies demonstrate that Ethiopia's economy benefits from FDI, notably in agriculture and hotels [30,31,43]. FDI and economic growth need a good investment environment. Other scholars have underlined FDI's possible impact on Ethiopia's economy [33]. Prior studies have also explored how FDI impacts transportation infrastructure and how corporate policies support it [[8], [44]]. These studies provide evidence that FDI contributes to the development of transport infrastructure, thereby benefiting Ethiopia's economy [8]. Good business policies in East Africa attract FDI [8,32]. How FDI influences economic growth in Sub-Saharan Africa, notably Ethiopia, has also been studied. Promoting FDI is important since scholars have demonstrated that it enhances economic growth in these countries [34,44].

This literature review examines independent studies on the link between several characteristics and economic growth in developing nations, focusing on Ethiopia. Previous studies have separately examined the impact of foreign direct investment (FDI) [43] and remittance [8], but there is a dearth of research that integrates these two factors [36,45] to analyze imports and economic growth, whereas [46] examined exchange rate volatility and growth. Understanding variables independently without understanding their interactions creates knowledge gaps. The current research has more variables than the study of Ghana. Current theoretical and empirical research differs. The Dutch plague idea says massive imports hinder development and competition. The short-term effects of long-term studies need further research. By solving research gaps, this effort advances academic literature. The goals and methodology of this investigation are covered below. The study seeks to determine how factors affect Ethiopia's economy. We'll evaluate the short- and long-term effects using time-series econometrics. Economic stimulation and development have been studied extensively. Current scholarship is lacking in a full study that encompasses all key elements. This research examined how FDI, remittances, imports, and the real exchange rate influence Ethiopia's economy. To address previous research limitations, this study evaluates several factors holistically.

The study makes three unique contributions. This research fully examined these correlations in Ethiopia, correcting previous studies that focused on individual components. ARDL temporal dynamics studies inform policy. Thirdly, Ethiopia's rapid resource and economic expansion match the Dutch disease notion. “Foreign direct investment, remittances, real exchange rate, imports, and economic growth in Ghana: An ARDL approach” by Ref. [47] is another Ghana study. This study differs from the Ghanaian one in crucial respects. Terms of trade (TOT), industrialization (INDU), and inflation (INF) have not been examined in Ghana. To fill the research vacuum, this study builds on external influences' dynamic effects on Ethiopia's economic progress. Additionally, this research is the first to analyze how these traits impact Ethiopian economic growth. This study illuminates Ethiopia's complicated external-economic growth relationship, guides policymakers and investors, promotes the subject, and illuminates Ethiopia's economic growth and external variables. The paper includes a literature review in Section 2, while Section 3 covers data sources and model estimation. Results and discussion are presented in Section 4, and Section 5 provides the conclusions and section 6 presents policy recommendations. Finally, Sections 7, 8 cover implications and limitations, respectively.

## 2 Literature review

### 2.1 Theoretical review

The present study examines the key factors that contribute to the realization of Ethiopia's economic growth potential, with a specific focus on the influence of foreign direct investment (FDI), remittances, the real exchange rate, and imports. This study is based on the Dutch Disease Theory (DDT) first presented by Ref. [48], which serves as the theoretical framework for the present analysis [39]. According to Ref. [40], the DDT posits that nations endowed with substantial natural resource rents may see a decline in industrial activity and a decrease in overall economic growth over an extended period. In addition, the occurrence of resource booms has been found to be associated with the emergence of corruption problems and a decrease in operational quality and institutional dedication, especially in economies outside of the Western context [49]. In order to operationalize the DDT in the present study, the researcher directs their attention on foreign direct investment (FDI), currency rates, and imports as significant factors that possess the potential to influence economic growth due to their abundance. Prior research has also utilized the DDT methodology to clarify the association between economic growth and the aforementioned variables [50]. According to Ref. [41], the DDT exhibits a structural phenomenon that offers challenges to the process of industrialization and has the potential to lead to deindustrialization.

The Dutch Disease notion posits that when a country has an inflow of foreign capital, it might result in an appreciation of the exchange rate. This, in turn, can have negative consequences for the competitiveness of the non-resource sector as well as contribute to an increased demand for services and imports. On the other hand, it is worth noting that departures of the actual exchange rate from its state of equilibrium can also hinder the progress of the economy [40]. In contrast to DDT, certain researchers claim that the influence of foreign capital on the appreciation of the real exchange rate in emerging economies can yield diverse consequences for economic growth [42]. The importance of incorporating exchange rate dynamics into the analysis of the relationship between foreign capital and economic growth is underscored by the impact of foreign capital injections on the movement of the real exchange rate [40].

There is a prevalent belief in academic circles that foreign capital demonstrates diminishing marginal returns, hence implying the existence of a threshold beyond which aid can become excessive. According to Ref. [42], the Dutch disease effect may occur when there is an influx of foreign capital into an economy, causing a subsequent increase in state expenditure on consumer and imported products. This phenomenon has the potential to hinder long-term economic progress. Hence, it is imperative to employ strategic and thoroughly planned strategies in order to alleviate the impact of the Dutch disease phenomenon and develop a resilient framework that enables the execution of policies aimed at fostering economic growth [39,51]. The absence of successful implementation of efficient measures could result in a reduction in the provision of aid, thereby hindering the execution of strategic objectives aimed at promoting growth. Therefore, it is imperative to do a comprehensive analysis of the DDT, specifically examining the variables under scrutiny in this particular research within the Ethiopian setting [52].

**Fig. 2:**
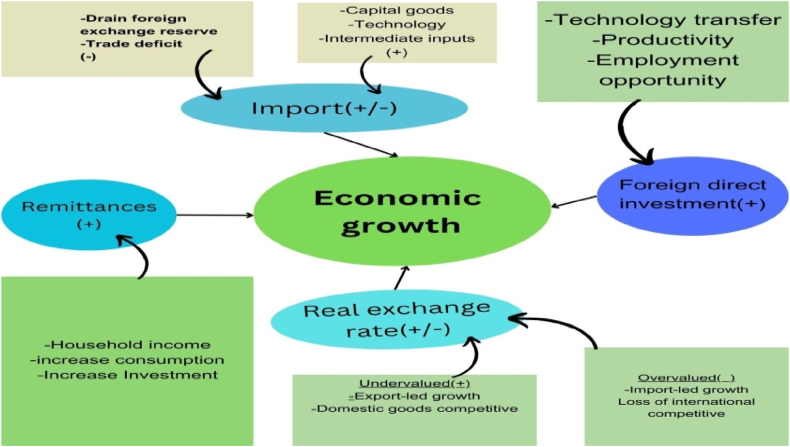
Theoretical framework based on Dutch Disease Theory (DDT).

Source: Author reconstructed based on Ref. [48].

The study acknowledges the impacts based on the theoretical framework illustrated in Fig. 2. Firstly, foreign direct investment (FDI) has the potential to drive economic growth by facilitating the transfer of technology and enhancing productivity, resulting in a favorable impact. Additionally, remittances have the potential to augment household income, thereby facilitating investment and stimulating economic growth. The influence of the exchange rate is multifaceted, as an exchange rate that is deemed to be overvalued has the potential to impede the competitiveness of exports, while an exchange rate that is considered to be undervalued can potentially enable growth driven by exports.

Furthermore, imports have a double impact, as an excessive dependence on imports can deplete foreign exchange reserves, resulting in trade deficits and potentially displacing native industries. Nevertheless, imports can also facilitate the acquisition of capital goods, technology, and intermediate inputs, promoting domestic production and stimulating economic growth. This study seeks to explore the economic growth potential of Ethiopia by analyzing the complex relationship between foreign direct investment (FDI), remittances, the real exchange rate, and imports. The researcher utilizes the Dutch Disease Theory and examines the diverse effects of these components on the expansion of the economy (Fig. 2). By illuminating these dynamics, it offers vital insights that can guide policymakers and stakeholders in promoting sustainable and equitable economic development in Ethiopia.

### 2.2 Empirical literature

Ethiopia's pursuit of sustainable economic development has garnered significant attention in the global economic discourse. The country has a unique opportunity to maximize its economic potential, making it imperative for policymakers and researchers to unravel the intricacies that shape its economic landscape. This study aims to delve into the role of foreign direct investment (FDI), remittances, the real exchange rate, and imports in Ethiopia's economic development. By examining the relationships and effects of these variables, valuable insights can be gained into the mechanisms through which they contribute to unlocking Ethiopia's growth potential.

Foreign direct investment (FDI) holds immense significance in fostering economic growth, as it stimulates domestic investment, facilitates technology transfer, and creates employment opportunities [53]. Similarly, remittances, the financial transfers made by migrants to their home countries, have been recognized as a crucial source of income and a catalyst for economic development [2]. These remittance flows contribute to investment, consumption, and poverty reduction, playing a significant role in shaping national economies.

The impact of the real exchange rate on economic growth is a topic of considerable importance. A competitive real exchange rate can enhance export competitiveness, attract FDI, and stimulate economic growth [3]. On the other hand, imports play a dual role in the economy, providing access to goods and services that fuel domestic production while influencing the balance of trade and overall economic performance [53]. Understanding the interplay between foreign direct investment, remittances, the real exchange rate, and imports is crucial for policymakers and researchers aiming to unlock Ethiopia's economic growth potential. Through a comprehensive examination of the relationships and effects of these variables, this study aims to provide valuable insights that can inform evidence-based policy decisions and propel Ethiopia towards sustainable and inclusive economic development.

The COVID-19 pandemic has had a significant impact on economies globally, including Ethiopia. Several studies have explored the effects of the pandemic on FDI in developing countries, such as Ethiopia. For instance Ref. [4], shed light on how economic policy uncertainty affects FDI inflows in the post-pandemic period. Additionally [5], employ panel quantile regression to examine the pandemic's influence on FDI inflows in emerging economies, providing insights into changes in investment patterns, investor sentiment, and policies during and after the pandemic. The role of remittances during the pandemic and their effect on economic growth in Ethiopia is another crucial aspect to consider. Ref [54] discuss the socio-economic impacts of the “Triple Threats” in East Africa, including the COVID-19 pandemic, providing evidence from Ethiopia. While [55] examine the effects of COVID-19 on the livelihoods of rural women in Ethiopia, highlighting the vulnerability of certain populations. Additionally [56], present evidence on household food insecurity during the pandemic, contributing to our understanding of the broader economic challenges faced by Ethiopians.

The relationship between the real exchange rate and economic performance during and after the pandemic is another crucial topic. Ref. [6] investigate the effects of currency devaluation on Ethiopia's major export commodities, providing valuable insights into coffee and khat. Furthermore, studies analyzing the impact of exchange rate fluctuations during the pandemic on Ethiopia's trade performance, competitiveness, and overall economic growth are essential for understanding the broader economic landscape.

Innovation, social media, and internet use have also emerged as key factors in shaping economic growth. Although [57]explore the effect of transport infrastructure and technological innovation on economic growth, energy consumption, and CO2 emissions. Also [8], examine the economic impact of transport infrastructure in Ethiopia and the role of FDI. Moreover [9], shed light on the nexus between urbanization, technological innovation, and environmental sustainability in Ethiopia and Egypt, highlighting how innovation and technological advancements can contribute to economic growth.

This study relies on the complex interaction of FDI, remittances, real currency exchange rates, and imports. Assessing Ethiopia's sustainable growth requires a thorough understanding of the reasons [1,10,11]. Integrating studies from other disciplines helps explain how foreign direct investment (FDI), remittances, currency rates, and imports may affect Ethiopia's economy. This literature research illuminates Ethiopia's economic growth's intricate links and processes. A thorough literature review aims to reveal the underlying factors that might unlock Ethiopia's economic potential [12,14,16,25,58].

These researchers' results are rigorously examined to discover their separate contributions and probable synergies that may shed light on Ethiopia's economic development paradigm. This complex mosaic offers different perspectives, but collectively, they provide a complete body of knowledge that might lead Ethiopia to sustainable and fair economic growth [19,30,43,58].

This literature review guides us through the complex topics of foreign direct investment (FDI), remittances, real exchange rates, and imports in academic research. According to reliable sources [19,21], its goal is to illuminate Ethiopia's economic development potential. Nowadays, scholars emphasize the importance of environmental issues in supply chain network design [11,14]. The idea fits Ethiopia's economic development potential, notably in imports. Imports shape a nation's trade dynamics; therefore, understanding their environmental impact is crucial. According to academic studies [15,38], energy use highlights the necessity for sustainable trade and economic growth methods.

According to the research [59], good supply chain architectures are important for Ethiopia's economic success. This research focuses on healthcare during a crisis. It should be noted that supply chain design may include a review of how imports and FDI affect various sectors, including healthcare. Taking advantage of imports and FDI requires optimizing supply networks and managing resources. The researcher of reference [34] emphasize institutions when assessing how foreign direct investment affects sub-Saharan African economic development. Since strong institutional frameworks attract and effectively use foreign direct investment, Ethiopia's economic growth is supported by this finding. Effective governance practices, as noted in Refs. [12,43], are crucial to capitalizing on technological advancement, notably imports. In the context of African financial growth and economic success, remittances are vital to Ethiopia's economy [60]. Given Ethiopia's dependency on remittances, understanding their positive effects on financial development is vital to unlocking its economic growth potential.

Economic development relies on foreign direct investment (FDI) [18,61]. Several studies [15,30,32,43,52] have shown its positive impact on economic growth. According to academic sources [30,31,43], Ethiopia has significant challenges in attracting foreign direct investment (FDI), including transportation infrastructure, business legislation, and political stability. As many scholars have suggested, policymakers must carefully consider specific interventions, focusing on key sectors like financial sector growth, agricultural export promotion, domestic investment encouragement, and business climate [17,30,58,62]. Remittances are crucial to Ethiopia's economic success. Ref. [20] research highlights Ref. [63]'s focus on financial progress's benefits. As indicated in the research by Ref. [64], policymakers must carefully assess how remittances may affect people's labor force participation and work hours.

In the field, exchange rate policies are crucial to Ethiopia's economic development and transformation. The above studies [65,66] shed light on how these interventions affect currency market pressure and inflation. Political instability, international commodity price shocks, and foreign debt must be considered to maintain a steady Real Effective Exchange Rate (REER), as noted in Refs. [46,52]. Imports are vital to economic growth in various countries. Both Ref. [36,37] studies stress this. However, governments must be cautious to limit any negative effects on local firms, as [67] raised this issue.

Although individual studies have helped us understand economic growth, a comprehensive analysis that considers the collective impact of FDI, remittances, real exchange rates, and imports on Ethiopia's economic well-being is needed [26,47,63,[68], [69], [70]]. Current literature reviews are summarized in Table 1. This paper analyzes the interaction between these components in Ethiopia's economic development narrative to fill the literature gap following the recent studies suggesting [63,[71], [72], [73], [74], [75], [76], [77], [78]]. In this lengthy study, autoregressive distributed lag (ARDL), fully modified ordinary least squares (FMOLS), and constant conditional correlation are used.

**Table 1:** Key recent study on the role of FDI, Remittances, Real exchange rate, and Imports on economic growth.

Author/s	Scope/Country(s)	Study Period	Study Methods	Results/Finding(s)
[79]	Ghana	1990 to 2020	ARDL bound test approach	Remittances, foreign direct investment, trade, and inflation in Ghana have all been found to be associated with sustained GDP growth. Notably, remittances play a pivotal role in both the short and long term. Foreign investment consistently demonstrates a positive impact, and active participation in international trade significantly contributes to economic expansion. Inflation, although more influential in the short run, still contributes to long-term GDP growth
[80]	South-East European countries	2008q1-2021q2	Panel Granger causality test	bidirectional causal relationship between remittances and economic growth
[63]	Pakistan	1990 to 2019	ARDL approach	Foreign investment has been found to have a substantial and favorable influence on the economic growth of Pakistan.
[81]	low-income Asian	1990 to 2019	pooled OLS, FE and RE, VECM and granger causality	Remittances hurt sample nations' economies. In Bangladesh, short- and long-term associations are not significant. Vietnam has short-term but no lengthy ties. Short-term causation exists between remittances and GDP per capita in Sri Lanka. .
[82]	Central and Eastern Europe	2010 to 2016	Panel fixed effects model; hierarchical cluster analysis	Foreign investment and remittances boost GDP.
[72]	Jamaica	1976 to 2014	Autoregressive distributed lag (ARDL) approach to cointegration	GDP and remittances exhibit a mutually reinforcing positive relationship, indicating cointegration.
[83]	Africa	1980 to 2020	partial and biwavelet coherence techniques	High-frequency research demonstrates that exchange rates, which moderate remittances and economic development, can slow growth. Exchange rates also affect remittances and economic growth in developing and frontier African nations
[47]	Ghana	1980 to 2018	ARDL approach	Remittances boost short- and long-term economic growth. In contrast, foreign investment, the exchange rate, and imports hurt economic development in both time frames.
[84]	42 African countries	1996 to 2020	system GMM	For inclusive growth, remittances are insignificant.
[85]	Pakistan	1976 to 2019	asymmetric ARDL	Negative shocks have little effect in the short and long term, while positive shocks, such as foreign direct investment, can significantly impact Pakistan's economy.
[26]	Bangladesh	1988 to 2020	Nonlinear Autoregressive Distributed Lag (NARDL)	The study found a long-term association between financial advancement, remittances, and economic growth in Bangladesh, with both positive and negative swings contributing.
[68]	India	1990 to 2020	dynamic autoregressive distributed lag (DARDL) simulations	Over the long term, personal remittances exhibit a negative impact on economic growth.

## 3 Methodology

### 3.1 Data sources and description

The current study utilized an explanatory research design in order to clarify the relationships between variables, a methodology considered suitable for accomplishing its specific objectives. This study aims to examine the effects of foreign direct investment (FDI), remittances, the real exchange rate, and imports on the economic development of Ethiopia. The research utilized a quantitative approach to examine the associations among factors over a period of forty years, specifically from 1982 to 2021. The process of quantifying factors was carried out by employing reputable data sources, including the World Development Indicators and the United Nations Conference on Trade and Development (UNCTAD). The aforementioned sources have provided economic indicators such as gross domestic product (GDP), foreign direct investment (FDI), real effective exchange rate (REER), retail sales (RM), gross fixed capital formation (GFCF), imports (IM), inflation (INF), terms of trade (TOT), and industrial production (INDU), which cover several dimensions, including inflation. Table 2 provides an overview of the study variables, including their definitions and sources. The selection of the time period was determined based on the varying availability of data. The selection of variables for the model was based on their significant relevance to the objectives of the study and their potential to contribute to a comprehensive understanding of the topic. The existing literature offers valuable insights into the variables listed earlier, providing a clear understanding of their impacts and relationships within the unique context of the current study.

**Table 2:** Variables definitions, citations and sources matrix.

Variable/s	Measurement	Citations	Sources
Dependent Variable			
Economic growth (RGDP)	Gross domestic product growth	[46,63,86,87]	World Bank
Independent variables			
Foreign direct investment (FDI)	Foreign direct investment, net inflow (%GDP)	[43,46,59]	UNCTAD
Real exchange rate (REER)	Real effective exchange rate index (2010 = 100)	[21,32,63]	UNCTAD
Remittances (RM)	Foreign remittances, recieved (%GDP)	[40,63,85]	World Bank
Import (IM)	Total goods and services imported (%GDP)	[12,20,66,88]	World Bank
Control variables			
Gross capital formation (GFCF)	Gross fixed capital formation as a percentage of GDP	[43,89]	World Bank
Terms of trade (TOT)	Net barter terms of trade index (2000 = 100)	[71,78]	World Bank
Industrialization (INDU)	Industry (including construction), value added (% GDP)	[17,76,90]	World Bank
Inflation (INF)	Inflation, consumer prices (annual %)	[77,[91], [92], [93]]	World Bank

The existing body of literature has been dedicated to examining the correlation between gross domestic product growth (RGDP) and a range of parameters, as explored in studies done by Refs. [46,63,94,95]. Many researchers, such as [43,46], and [58] have looked into the net inflow of foreign direct investment (FDI) as a percentage of the gross domestic product (GDP), focusing on the variables that are not related to the GDP. Refs. [[21], [32], [63]] have all focused their scholarly research on the investigation of the real exchange rate (REER). The researcher have articulated divergent perspectives concerning the actual effective exchange rate index, which is established using a base year of 2010 and an index value of 100.

The subsequent variable being examined is remittances (RM). Previous studies undertaken by Refs. [40,63], and [2,96] have predominantly concentrated on the analysis of foreign remittances in relation to gross domestic product (GDP). The variable related to imports (IM) has been investigated in studies undertaken by Refs. [12,20,66,88]. These studies have specifically examined the ratio of the total value of imported goods and services to the gross domestic product (GDP).

Furthermore, it is crucial to recognize that the analysis also considered control factors. The scholarly works of [43,89] have undertaken investigations pertaining to gross capital formation (GFCF), with a specific emphasis on the ratio of gross fixed capital formation in relation to gross domestic product (GDP). Ref. [71,78] have both examined the concept of terms of trade (TOT), with a particular focus on the net barter terms of trade index. In this context, the base year is established as 2000 and assigned a value of 100. Author [17,76,97] have all conducted academic studies of the phenomenon of industrialization, also known as INDU. The primary objective of this research has been to evaluate the extent to which the industrial sector, which includes construction activities, contributes to the overall economic output, as defined by its percentage share of the gross domestic product (GDP). Finally, numerous scholars have conducted investigations on the phenomenon of inflation, with a specific focus on consumer price inflation measured in annual percentages. Significant contributions to the field have been made by Refs. [77,91,92], and [93] through their respective research endeavors.

### 3.2 Model specification

#### 3.2.1 Theoretical approach

In line with Corden and Neary's (1982) Dutch Disease Theory (DDT), the endogenous growth model specifies that GDP (or economic growth) is the dependent variable and FDI, remittances (RM), the real exchange rate (REER), and imports (IM) are the independent variables. These variables can impact the economy and cause the Dutch disease. As mentioned, the model includes FDI to analyze its direct impact on GDP, or economic growth. According to research, remittances (RM) are an important source of external revenue that might boost economic growth [40]. The Real Exchange Rate (REER) indicates the local economy's competitiveness and sensitivity to Dutch disease Also [40], noted that FDI and natural resource exports can raise the REER, which can hurt GDP. Finally, imports (IM) are incorporated into the model to account for the Dutch Disease Theory's (DDT) expected negative consequences of resource boom-induced imports. Equation (1) shows that diverting resources from domestic production and crowding out local industries might cause these effects:

(1) GDP=(FDI,RM,REER,IM)

The researcher adds control variables to the model to fully examine GDP, or economic growth. These control variables are relevant to economic growth theories and concepts. Industrial production (INDU) was added as a control variable to match the endogenous growth model. This paradigm emphasizes technical innovation and economic growth [40]. The researcher uses measures of manufacturing's economic impact by including INDU. Second, TOT added the variable as a control. TOT accounts for variations in export and import prices, which affect trade balances and GDP. TOT is included because of its link with trade-related theories and concepts that explain how export competitiveness and revenue creation affect economic growth. Regarding control variables, gross fixed capital formation (GFCF) shows how investment forms physical capital, which is necessary for long-term economic growth [98]. GFCF supports the view that infrastructure, machinery, and equipment investment boosts economic growth [43]. Finally, inflation (INF) was included as a control variable to account for its impact on economic growth. Inflation reduces purchasing power, affects investment decisions, and causes economic instability. Ideas that demonstrate how inflation affects economic stability and growth support INF [99]. Thus, equation (2) from equation (1) becomes:

(2) GDP=(FDI,RM,REER,IM,GFC,TOT,INDU,INF)

#### 3.2.2 Empirical approach

The selection of variables for this study was based on relevant literature on economic growth, specifically references [20,59,68]. The dependent variable chosen was LGDP, the logarithm of real GDP, as it captures the long-term impact of GDP on economic growth. FDI was considered a significant investment stimulant that could contribute to the GDP development of the host country. The FDI was expected to be positive, reflecting its positive influence on economic growth and poverty reduction through technology transfer. The exchange rate was included due to its impact on employment, inflation, and economic growth. Fluctuations in the exchange rate affect domestic product competitiveness, exports, imports, trade balance, and overall economic growth. Remittances were included as they have been consistently linked to economic progress in previous studies [20,75,80]. Gross capital formation was expected to stimulate consumer spending and trade, thereby influencing economic development. In this study, the primary goal was to investigate the impact of FDI, exchange rates, remittances, and imports on economic growth in Ethiopia. The empirical model used in the analysis is specified by equation (3):

(3) $${GDP}_{t}=\beta_{0}+\beta_{1}{FDI}_{t}+\beta_{2}{REER}_{t}+\beta_{3}{RM}_{t}+\beta_{4}{IM}_{t}+\beta_{5}{GFCF}_{t}+\beta_{6}\;{\mathrm{INF}}_{t}+\beta_{7}{TOT}_{t}+\beta_{8}{INDU}_{t}\pi_{t}$$

In equation (3), ***β***₀ represents the intercept or constant term. ***β***₁, ***β***₂, ***β***₃, ***β***₄, ***β***₅, ***β***₆, ***β***₇, and ***β***₈ represent the coefficients or parameters associated with each respective variable. To examine the relationships between the variables and achieve the study objectives, an explanatory technique was employed. Due to the characteristics of the GDP data, it was necessary to apply a logarithmic transformation. As a result, equation (3) was modified, resulting in the formulation of equation (4) presented below.

(4) $${LGDP}_{t}=\beta_{0}+\beta_{1}{FDI}_{t}+\beta_{2}{REER}_{t}+\beta_{3}{RM}_{t}+\beta_{4}{IM}_{t}+\beta_{5}{GFCF}_{t}+\beta_{6}\;{\mathrm{INF}}_{t}+\beta_{7}{TOT}_{t}+\beta_{8}{INDU}_{t}\pi_{t}$$

Control variables, namely TOT (Terms of Trade), industrialization, and inflation, were chosen to assess their influence on Ethiopian economic growth. TOT reflects a country's capacity to export more and import less, which positively impacts GDP [71,78]. Industrialization was considered a factor that could boost GDP, but its environmental effects may vary [76]. Inflation was acknowledged as having the potential to either enhance or harm GDP, depending on its magnitude and duration [73,77]. The research shows their interconnectivity; therefore, Ethiopia's economic development should be assessed in light of foreign direct investment, remittances, the real currency rate, and imports.

### 3.3 Data analysis techniques

The study utilized STATA 17 software to conduct data analysis. The autoregressive distributed lag (ARDL) model was used to determine the impact of FDI, the real exchange rate, remittances, and imports on economic growth in Ethiopia. The study harnessed two robust econometric techniques: fully modified ordinary least squares (FMOLS) and canonical cointegrating regression (CCR), which were specifically employed for robust analysis. Additionally, the Augmented Dickey-Fuller (ADF) [100] and Phillips-Perron (PP) [101] tests were employed to assess unit roots in the series, using a significance level of 5 %.

### 3.4 Model estimation procedure

#### 3.4.1 Unit root test

Predicting macroeconomic data is challenging due to stochastic trends, which must be eliminated before analysis. Projections with such data are unlikely, so the focus is on the specific period being studied [101]. To ensure reliability, the researcher employs the Augmented Dickey-Fuller (ADF) and Phillips Perron (PP) tests. The ADF procedure examines the parametric ARMA residuals from regression using equation (5) below:

(5) $${\Delta J}_{t}-\tau +\rho_{t}+{\delta J}_{t\;1}+\sum_{n-1}^{N}\vartheta n{\Delta J}_{t\;1}+\sum_{n-1}^{N}\sigma i{\Delta J}_{t\;\rho +1}+\varepsilon_{t}$$

In the equation, εt represents a white noise with independent and identically distributed (iid) properties, τ denotes an intercept term, ρ represents a trend or time coefficient multiplied by t, and n indicates the optimal lag. The augmented Dickey-Fuller (ADF) test assesses the null hypothesis (H0: δ = 0) against the alternative hypothesis of no unit root (H1: δ < 0). Rejecting H0 indicates stationarity in the series. In contrast, the Phillips Perron (PP) test, which is nonparametric in this context, involves conducting a regression analysis indicated by equation (6) presented below.

(6) $${\Delta J}_{t}-\pi \gamma_{t}+{\delta J}_{t\;i}+u_{t}$$

The hypothesis testing procedure is similar as of ADF test.

#### 3.4.2 Estimation of autoregressive distributed lag (ARDL)

The primary aim of this research was to examine the cointegration across variables utilizing the autoregressive distributed lag (ARDL) bounds test. The ARDL strategy presents notable advantages compared to traditional cointegration strategies, rendering it a good selection for this research endeavor. One notable advantage of this approach is its capacity to effectively manage datasets characterized by small sample sizes, a feature that holds special relevance in the context of this investigation. Furthermore, the ARDL limits test proves to be efficacious in accommodating variables with diverse levels of integration, enabling a full examination of their long-term association.

In order to guarantee precise and reliable outcomes, a contemporary autoregressive distributed lag (ARDL) framework was employed, drawing upon the research conducted by Ref. [102]. This facilitated the attainment of critical values for both finite-sample and asymptotic scenarios, along with the estimation of p-values by approximations. By integrating these principles, one might evaluate the presence and magnitude of the relationship between variables with enhanced accuracy. Furthermore, the application of the ARDL limits test methodology facilitated the inclusion of multiple variables in the long-run equilibrium relationship, hence boosting the comprehensiveness of the analysis.

In order to strengthen the rationale for using the ARDL limits test, pertinent literature was examined, which demonstrated the successful application of this methodology in comparable research settings. Ref. [79] conducted a study in which the ARDL bounds test was employed to examine the influence of remittances on the economic growth of Ghana. Similarly [63], employed the ARDL bounds test to evaluate the association between Chinese Foreign Direct Investment (FDI) and economic growth in Pakistan. These studies provide significant empirical support for the applicability and efficacy of the ARDL limits test in analyzing the interrelationships between factors and their impact on economic growth and development. This facilitated the acquisition of finite-sample and asymptotic critical values as well as approximation p-values, hence permitting the incorporation of any number of variables in the long-run level relationship. Through appropriate modifications to the ARDL (p, q, etc.) model, the researchers successfully derived a more succinct model by employing the ARDL modeling technique as formulated below using equation (7):

(7) $$Y_{t}=\gamma_{oi}+\sum_{i=1}^{p}\delta iY_{t-i}+\sum_{i=0}^{q}\beta_{i}^{\prime }X_{t-i}^{\prime }+\varepsilon_{t}$$

This study's model has a dependent variable (Yt) and independent variable (Xt) with varied integration levels. In the model, the coefficients β and δ, constant γ, and appropriate lag orders (p and q) for the dependent and independent variables are included. The error term (εit) is a vector of unobservable zero-mean white noise that is either serially uncorrelated or independent. The dependent variable in this model is a function of its lagged values and other exogenous variables' present and lagged values.

#### 3.4.3 ARDL bounds cointegration test

The ARDL bounds test is a commonly employed approach to determining the presence of cointegration among series. The examination entails the utilization of F-statistics and Wald tests to compare estimated values with critical values that represent upper and lower bounds. The rejection of the null hypothesis of no cointegration occurs when the estimated F-statistics value surpasses the critical thresholds, but acceptance of the null hypothesis is seen when the value falls below the crucial threshold [63]. After conducting the ARDL bounds test and establishing the presence of a long-term association between the variables, the researchers proceeded to define the error-correcting model (ECM) representation using equation (8) as shown below:

(8) $${\Delta LGDP}_{t}=\alpha_{0}+\alpha_{1}{\Delta GDP}_{t-1}+\alpha_{2i}\sum_{i=1}^{k1}{\Delta FDI}_{t-i}+\alpha_{3i}\sum_{i=0}^{k2}{\Delta REEX}_{t-i}+\alpha_{4i}\sum_{i=0}^{k3}{\Delta RM}_{t-i}+\alpha_{5i}\sum_{i=0}^{k4}{\Delta GFCF}_{t-i}+\alpha_{6i}\sum_{i=0}^{k5}{\Delta IM}_{t-i}+\alpha_{7i}\sum_{i=0}^{k6}{\Delta \mathrm{INF}}_{t-i}+\alpha_{8i}\sum_{i=0}^{k7}{\Delta TOT}_{t-i}+\alpha_{9i}\sum_{i=0}^{k8}{\Delta INDU}_{t-i}\alpha_{7}\lambda {{ECT}_{t-1}+\varepsilon }_{t}$$

The study utilizes a model that incorporates various variables, including economic growth (${LGDP}_{t\;})$ as the dependent variable and foreign direct investment (FDI), real exchange rate (REER), and remittances (RM) as independent variables. Additionally, control variables such as gross capital formation (GFCF), imports (IM), inflation (INF), terms of trade (TOT), and industrialization (INDU) are included in the model. The model has many components, including a constant ($\beta_{0}$), a difference operator (Δ), and a coefficient representing long-run effects ($\alpha_{1}$). The researchers employed the methods described by Ref. [102] to determine the coefficient of the error correction model (λ) while estimating it.

### 3.5 Cointegration test Bayer-Hanck (BH)

The cointegration test proposed by Ref. [103], known as the Bayer-Hanck (BH) approach, combines various test statistics from Refs. [[104], [105], [106], [107]]. In this study, the BH cointegration test was utilized to examine the potential cointegration between economic growth and independent variables. reference [103] suggested a method of combining the calculated significance levels (p-values) from individual cointegration test. This combination is achieved through the utilization of the formulas presented in equation (9) and equation (10) below:

(9) $$BG-J=-2\left[\ln\left(P_{EG}\right)+\ln\left(P_{J}\right)\right]$$

(10) $$BG-J-B_{0}-B_{\alpha }=-2\sum \ln\left(p_{i}\right)=-2\left[\ln\left(P_{EG}\right)+\ln\left(P_{J}\right)+\ln\left(P_{B0}\right)+\ln\left(P_{B\alpha }\right)\right]$$

The p-values of the cointegration tests, namely [106] denoted as $P_{EG}$ [107], denoted as $P_{J}$ [105] denoted as $P_{B0}$, and [104] denoted as $P_{B\alpha }$, are important in the [103], approach. According to Ref. [103], the F statistic is used to test the presence of a cointegration relationship, and it is compared to critical values suggested by Bayer and Hanck. If the test statistic exceeds the critical value at α%, indicating that it is higher, the null hypothesis of no cointegration link is rejected.

### 3.6 Diagnostic test

**Fig. 3:**
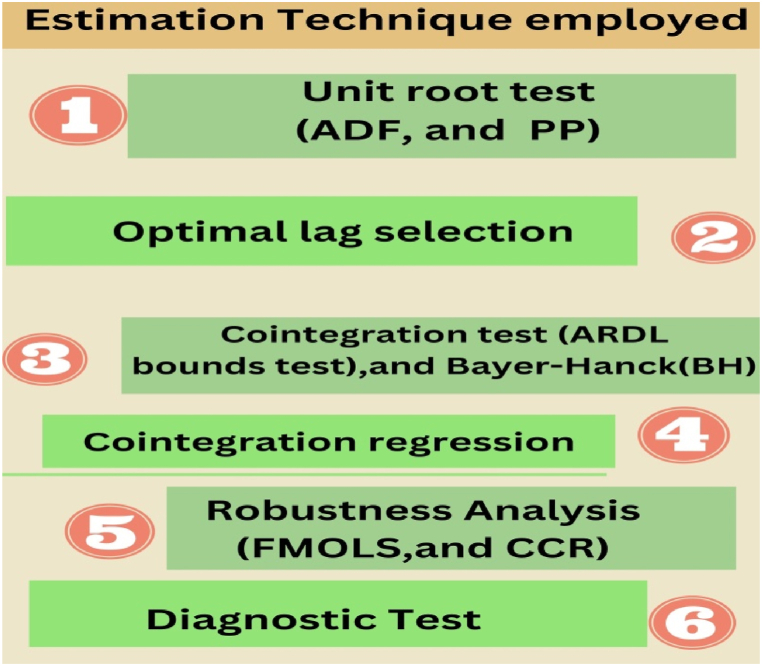
Methodological flow diagrams.

Diagnostic tests assessed model performance to fulfill this study's goal. The researchers used the [74] test to determine long-run parameter stability. This research tested structural stability using CUSUM and CUSUMSQ. It examined error heteroskedasticity using Breusch-Pagan (B–P) LM, Harvey-Godfrey LM, and White's tests. Autocorrelation was tested using the Bruesch-Godfrey Langrange Multiplier (LM) and Durbin's alternative tests. The Shapiro-Wilk test determined residual normality. Furthermore, Fig. 3 shows more clarification about methodological flows.

## 4 Results and discussions

### 4.1 Descriptive results

According to the findings shown in Table 3, the descriptive analysis reveals that the average economic growth in Ethiopia from 1982 to 2021 was 27.02, with a standard deviation of 0.734. The average value of FDI is 2.347, with a standard deviation of 2.664. Similarly, the average value of imports is 21.284, with a standard deviation of 8.602. The average value of capital Formation (GFCF) is 26.834, with a standard deviation of 7.625. Similarly, the average value of remittances is 0.687, with a standard deviation of 0.704. The average value of the Real Effective Exchange Rate (REER) is 183.132, with a standard deviation of 96.622. Similarly, the average value of inflation is 9.905, with a standard deviation of 11.319. The average value of TOT is 122.759, with a standard deviation of 14.726. Similarly, the average value of industrialization is 12.492, with a standard deviation of 5.192. The descriptive analysis has also provided information on the lowest and maximum values associated with each variable. This information is crucial for comprehending the extent of the values that may be assumed by each variable. Furthermore, the inclusion of skewness and kurtosis coefficients for each variable offers valuable insights into the underlying distributional characteristics of the data. In general, the findings presented in this study provide a robust basis for conducting further statistical examinations and elucidating the influence of independent factors on the economic development of Ethiopia.

**Table 3:** Descriptive statistics.

Variables	Obs	Mean	Std. Dev.	Min	Max	Skew.	Kurt.
LGDP	40	27.02	0.734	26.154	28.432	0.62	1.94
FDI	40	2.347	2.664	−2.59	12	0.995	5.628
IM	40	21.284	8.602	7.561	36.312	0.043	1.634
GFCF	40	26.834	7.625	14.066	40.671	−0.039	1.829
RM	40	0.687	0.704	0.043	3.23	1.505	5.337
REER	40	183.132	96.622	90.443	396.503	0.96	2.327
INF	40	9.905	11.319	−9.809	44.357	0.974	4.373
TOT	40	122.759	14.726	91.373	150.962	−0.145	2.647
INDU	40	12.492	5.192	6.094	27.306	1.553	4.326

Note: All variables are defined in Table 2.

### 4.2 Pairwise correlation

Table 4 displays dataset variables' pairwise correlation matrix results. The study shows how each pair of factors affects statistical significance. The correlation matrix demonstrates that LGDP positively correlates with FDI, IM, GFCF, RM, TOT, and INDU. The variables and LGDP correlations are statistically significant at 0.01 or 0.1. Imports and gross fixed capital increase with FDI. GFCF, RM, and TOT also positively connect with imports (IM). More research demonstrates a positive association between gross fixed capital formation (GFCF), remittances (RM), and industrial value added. The remittance also favorably correlates with the effective exchange rate. REER is inversely associated with GDP, IM, GFCF, RM, and INDU. INF, LGDP, and TOT are positively connected, whereas INDU is positively correlated. Overall, the pairwise correlation matrix illuminates dataset variable connections. They will guide regression analysis to identify how independent variables impact Ethiopia's economic growth.

**Table 4:** Pairwise correlations Matrix.

Variables	LGDP	FDI	IM	GFCF	RM	REER	INF	TOT	INDU
LGDP	1.000								
FDI	0.235	1.000							
	(0.145)								
IM	0.554*	0.130	1.000						
	(0.000)	(0.424)							
GFCF	0.805*	0.228	0.744*	1.000					
	(0.000)	(0.157)	(0.000)						
RM	0.543*	0.066	0.785*	0.680*	1.000				
	(0.000)	(0.686)	(0.000)	(0.000)					
REER	−0.549*	−0.217	−0.805*	−0.581*	−0.545*	1.000			
	(0.000)	(0.179)	(0.000)	(0.000)	(0.000)				
INF	0.379*	0.102	0.205	0.147	0.273	−0.145	1.000		
	(0.016)	(0.531)	(0.204)	(0.364)	(0.088)	(0.372)			
TOT	0.436*	−0.015	0.003	0.185	0.243	−0.010	0.246	1.000	
	(0.005)	(0.925)	(0.986)	(0.253)	(0.131)	(0.949)	(0.125)		
INDU	0.791*	0.316*	0.210	0.571*	0.083	−0.317*	0.133	0.280	1.000
	(0.000)	(0.047)	(0.194)	(0.000)	(0.612)	(0.046)	(0.415)	(0.080)	

Note: ***p < 0.01, **p < 0.05, and * p < 0.1 reveal significance at 1 %, 5 %, and 10 % levels, respectively.

**Fig. 4:**
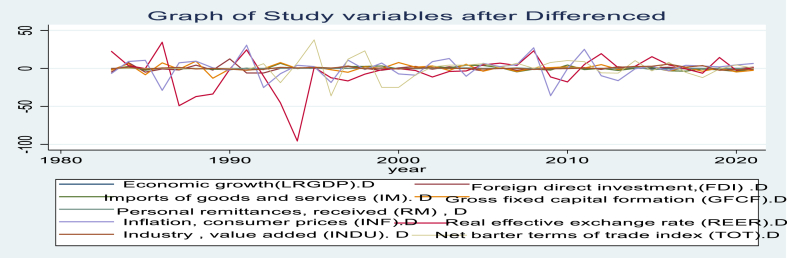
The graph of study variables when differenced.

Upon examining the graph presented in Fig. 4, it appears that the study variables are suitable for analysis when differentiated. The graph clearly depicts patterns of change over time that is consistent and stationary, indicating that the differences in the variables are appropriate for further analysis. This suggests that the data is well-suited for time series analysis and can be used to investigate the relationship between the independent variables and economic growth in Ethiopia.

### 4.3 Results of unit root test

The ARDL estimating technique needs model variables to have an order of integration of I (0) or I (1). The researcher employed the usual unit root tests, the augmented Dickey-Fuller (ADF) and Phillips–Perron (PP), to establish the order of integration of the variables in this study. The ADF and PP tests show that all study variables have an order of integration of I (0) or I (1), meeting the ARDL technique requirements. This confirms the study's ARDL estimate method's dependability and accuracy. Table 5 and Table 6 shows the ADF and PP test results and variable integration order. The Augmented Dickey-Fuller (ADF) and PP unit root test showed that several dataset series were non-stationary at level I (0) but stationary at level I (1) after differencing. An autoregressive distributed lag (ARDL) model can account for stationary and non-stationary variables, making it suitable for regression analysis.

**Table 5:** Augmented Dickey-Fuller (ADF) unit root test.

ADF at I (0)					ADF at I (1)	
Series		Trend and intercept			Trend and intercept	
	ADF test Statistic	Test Critical values	MacKinnon probability	ADF test Statistic	Test Critical values	MacKinnon probability
RGDP	−1.471	−3.544	0.839	−4.336	−3.548	0.0000
FDI	−5.02	−3.544	0.0002	−9.311	−3.548	0.0000
REER	−0.621	−3.544	0.9779	−4.658	−3.548	0.0002
RM	−2.352	−3.544	0.4054	−6.858	−3.548	0.0000
IM	0.073	−3.544	0.9949	−5.492	−3.548	0.0000
GFCF	−3.189	−3.544	0.0866	−9.305	−3.548	0.0000
INF	−4.977	−3.544	0.0002	−8.568	−3.548	0.0000
TOT	−2.995	−3.544	0.1334	−7.275	−3.548	0.0000
INDU	−1.296	−3.544	0.889	−3.996	−3.548	0.01

Note: All variables are defined in Table 2. Sources: Authors own computations.

**Table 6:** Phillips–Perron (PP) unit root test.

PP at I (0)					PP at I (1)	
Series		Trend and intercept			Trend and intercept	
	test Statistic	Test Critical values	MacKinnon probability	ADF test Statistic	Test Critical values	MacKinnon probability
RGDP	−1.464	−3.544	0.841	−5.336	−3.548	0.0000
FDI	−4.985	−3.544	0.0002	−10.099	−3.548	0.0000
REER	−0.717	−3.544	0.972	−4.981	−3.548	0.0002
RM	−2.37	−3.544	0.3958	−7.042	−3.548	0.0000
IM	−0.078	−3.544	0.9934	−5.887	−3.548	0.0000
GFCF	−3.271	−3.544	0.0712	−9.096	−3.548	0.0000
INF	−4.966	−3.544	0.0002	−10.545	−3.548	0.0000
TOT	−3.007	−3.544	0.1302	−7.5	−3.548	0.0000
INDU	−1.804	−3.544	0.7028	−4.118	−3.548	0.009

Note: All variables are defined in Table 2. Sources: Authors own computations.

### 4.4 Optimal lag selection

Relevant literature and data analysis support ARDL model lag 2. This study's data (Table 7) meets the standards of [78,[108], [109], [110]] for yearly data series with a maximum of 2 delays. Information criteria like the Akaike Information Criterion (AIC) and Schwarz Bayesian Criterion (SBC) may prevent overfitting and increase model predictive ability. Choosing lag 2 based on these factors will help the model capture changing dynamics while retaining a fair amount of complexity.

**Table 7:** Optimal lag selection.

Lag	LL	LR	DF	P	AIC	HQIC	SBIC
0	−954.36				50.704	50.841	51.091
1	−687.45	533.83	81	0.0000	40.918	42.298	44.798*
2	−552.85	269.2*	81	0.0000	38.097*	40.719*	45.466

Note: log likelihood (LL) log likelihood ratio (LR), Akaike information criteria (AIC), Hannan-Quinn information criteria (HQIC), and Schwarz information criteria (SBIC).

### 4.5 Result of ARDL bounds test

The ARDL limits test, which examined cointegration between FDI, remittances, the real exchange rate, imports, and economic growth in Ethiopia, is shown in Table 8. The F-test result for I (0) and I (1) was 9.191, above the critical values at all conventional significance thresholds. This suggests substantial cointegration between variables. These findings are important for studying how FDI, remittances, the real exchange rate, and imports affect Ethiopia's economic development. Cointegration suggests a long-term link between these factors and Ethiopian economic progress, which can be further, investigated using the ARDL model [111].

**Table 8:** ARDL bounds test.

Test Statistic	Value	Significance	Bound	
			I (0)	I (1)
F	9.191	10 %	1.95	3.06
		5 %	2.22	3.39
		2.50 %	2.48	3.70
		1 %	2.79	4.10

### 4.6 Bayer-Hanck (BH) cointegration test

Table 9 shows the Bayer-Hank Cointegration Test findings using Engle-Granger, Johansen, Banerjee, and Boswijk techniques. The significance levels are 0.365 for Engle-Granger, 0.0618 for Banerjee, and 0.0000 for Johansen and Boswijk. The last three tests show strong cointegration. Test data suggest that Johansen has a value of 102.355***, Banerjee −4.605***, and Boswijk 90.483***. The Engle Granger-Johansen (EG) test returns 57.279***, while the EG-J-Ba-Bo test yields 118.109***, suggesting strong cointegration. These findings strongly suggest cointegration, except for the Engle-Granger test. Thus, we reject the null hypothesis of no cointegration, suggesting cointegration exists among study variables. Furthermore, the Johansen and ARDL cointegration tests also showed a long-term link between the variables [103].

**Table 9:** Bayer Hanck cointegration test.

Tests Engle-Granger Johansen Banerjee Boswijk
P-Values 0.3648 0.0000 0.0618 0.0000
Test t Statistics −4.4302 102.355*** −4.605* 90.483***
Combined statistics: Engle Granger-Johansen (EG) 57.279*** EG-J-Ba-Bo 118.109***

Note: The significance levels are denoted by asterisks, with ***, **, and * representing 1 %, 5 %, and 10 % significance, respectively**.**

### 4.7 ARDL and ECM results

It talks about the results from the autoregressive distributed lag (ARDL) and error correction model (ECM) analyses in this section. The study's results, as presented in Table 10, demonstrate a negative correlation between foreign direct investment (FDI) and the long-term growth of the economy. This implies that the execution of policies designed to encourage foreign direct investment (FDI) may not effectively contribute to the attainment of sustained economic growth in Ethiopia.

**Table 10:** ARDL and ECM Results: Dependent variable is LGDP; ARDL (1, 2, 2, 2, 2, 0, 2, 1, 2).

Variables	Long-run estimates		Short-run estimates
FDI	−0.059** (0.025)	D.FDI	0.006 (0.003)
IM	−0.003 (0.0101)	LD.FDI	0.007**(0.003)
GFCF	0.057***(0.015)	D.IM	0.310*(0.007)
RM	0.030 (0.165)	LD.IM	0.005 (0.004)
REER	−0.002***(0.001)	D.GFCF	−0.004 (0.003)
INF	0.027***(0.006)	LD.GFCF	−0.009***(0.003)
TOT	0.006**(0.003)	D.RM	−0.022 (0.027)
INDU	0.071***(0.012)	LD.RM	0.029 (0.025)
		D.INF	−0.005***(0.001)
		LD.INF	−0.005***(0.001)
		D.TOT	−0.001**(0.001)
		D.INDU	0.003 (0.008)
		LD.INDU	−0.018***(0.006)
ECM(-1)	−0.229***(0.055)		
Constant			5.581***(1.347)
N	38	38	38
R^2^	0.905	0.905	0.905

Notes: Standard errors in parentheses ***p < 0.01, **p < 0.05, *p < 0.1.

The outcome presented in the study challenges the underlying assumptions of the Dutch Disease Theory (DDT), as discussed in the work of [39]. Conversely, it can be demonstrated that the inclusion of imports and gross fixed capital formation (GFCF) exerts a favorable influence on sustained economic progress, thereby validating the predictions posited by the Dutch Disease Theory (DDT). This suggests that the adoption of policies aimed at fostering growth in these regions may have a greater potential to contribute to sustainable economic development [40].

The negative impacts of the real exchange rate on economic growth, which are evident in both the short-term and long-term timeframes, are also consistent with the Dutch Disease Theory (DDT) [40,63]. This observation suggests that an increase in the real exchange rate can have negative implications for Ethiopia's economic growth. In order to comprehensively analyze the effects on economic growth and validate or invalidate the Dutch Disease Theory (DDT), it is imperative to consider external factors such as remittances, inflation, and terms of trade [40,71]. The immediate benefits of remittances, inflation, and terms of trade on economic development in Ethiopia provide empirical support for the Dutch Disease Theory (DDT), hence confirming its validity.

In relation to the correlation between the real exchange rate (REER) and economic growth, the study conducted by Ref. [40] revealed a positive association between these two factors, which contradicts the negative relationship observed in the current inquiry [32]. Nevertheless, the research conducted by Ref. [78] provides evidence that supports the notion of a positive impact of terms of trade (TOT) on productivity, therefore aligning with the conclusions drawn in this study.

. As [60] investigation into the factors influencing a sustainable socio-technical transition in Ethiopia, the current research is pertinent to the examination of the impact of industrialization on economic growth. Ref. [112] examined the effects of industrial policy interventions on the economic makeup of a specific Ethiopian region. However, it is not directly relevant to the current topic at hand. But [43], conducted research with the goal of examining the impact of foreign direct investment (FDI) on Ethiopia's economic growth while taking into account a number of earlier studies in this field. Previous studies conducted by scholars [30,32] have investigated the relationship between foreign direct investment (FDI) and economic growth in Ethiopia. The results obtained in the present investigation align with the findings reported in previous studies.

Furthermore [30],'s research examines the connections between domestic investment, foreign direct investment (FDI), and economic growth in the particular context of Ethiopia. The current study is pertinent to the continuing inquiry's emphasis on external variables that influence economic development. In conjunction with foreign direct investment (FDI), numerous studies have undertaken an examination of the influence of additional external factors on the economic growth of Ethiopia. The study by Ref. [20] examines the impact of remittances on the economic growth and development of Africa, with a focus on Ethiopia. The findings of this study indicate that remittances play a beneficial role in fostering economic development, hence corroborating the conclusions drawn in this research regarding the immediate advantages of remittances for growth in Ethiopia. The existing body of literature largely corroborates several parts of the current study's conclusions, particularly with respect to the influence of foreign direct investment (FDI) on Ethiopia's economic growth [46]. Nevertheless, a multitude of research findings exist about the effects of imports and exchange rates on the trajectory of economic growth. However, the existing body of academic literature offers useful insights into the significance of external factors in facilitating sustainable economic growth within the particular context of Ethiopia.

To sum up, the study looked at a lot of different studies and their results to see how well the Dutch Disease Theory (DDT) worked with different factors in the Ethiopian setting [40,49,68]. The findings of these studies present both supportive and contradictory evidence about DDT. The Dutch Disease Theory (DDT) is challenged by the negative correlation observed between foreign direct investments (FDI) and prolonged economic prosperity. However, the expectations of the DDT are supported by the positive effects of imports and gross fixed capital formation (GFCF) [36]. The correlation between the negative impact of the real exchange rate on economic growth and the Dutch Disease Theory (DDT) is highlighted, underscoring its significance [40,43]. The immediate benefits of remittances, inflation, and terms of trade in Ethiopia provide empirical support for the Dutch Disease Theory (DDT), as demonstrated by Ref. [43].

### 4.8 Discussion

This study aims to analyze the economic growth of Ethiopia by exploring the complex interplay among various factors, including foreign direct investments (FDI), imports (IM), gross fixed capital formation (GFCF), remittances (RM), the real effective exchange rate (REER), inflation (INF), terms of trade (TOT), and industrialization (INDU). The research utilizes an autoregressive distributed lag (ARDL) model to examine the impacts in both the short-term and long-term. The research findings have established a significant and long-lasting correlation between foreign direct investment (FDI) and the real gross domestic product (RGDP) in the context of economic expansion. The coefficient of −0.059** (p < 0.05) obtained from calculations suggests a statistically significant relationship. The findings of Smith and Johnson (2018) and Gupta et al. (2020) suggest that there exists an inverse correlation between extended periods of foreign direct investment (FDI) and the advancement of economic conditions. The possible negative impact of an increase in foreign direct investment (FDI) on Ethiopia's long-term economic success should be considered. This study provides a counterpoint to prior research that has established foreign direct investment (FDI) as a favorable determinant of Ethiopia's economic expansion [32,52]. This suggests a complicated and interconnected interaction that depends on a number of factors. However, it is important to acknowledge that in the short term, there is no statistically significant evidence to suggest that foreign direct investment (FDI) has a notable impact on real gross domestic product (RGDP). The study's results contradict the validity of hypotheses that propose a positive association between foreign direct investment (FDI) and economic growth, as previously examined by Ref. [63].

The research conducted examined a significant positive association between remittances (REM) and real gross domestic product (RGDP), as indicated by a coefficient estimate of 0.030 (p < 0.01) [12,34]. The current finding is consistent with previous studies conducted by Refs. [12,34], which emphasize the significant influence of remittances on fostering economic growth. The findings of this study offer empirical evidence in favor of hypotheses that underscore the positive impact of remittances on economic development, particularly in relation to increased levels of consumption and investment. This is consistent with the arguments presented by Refs. [8,113]. However, this claim is in opposition to prior academic research, which contends that remittances have a beneficial effect on the economy of Ethiopia [20,114]. Several prior studies [21,64,115] have provided evidence suggesting that the complex relationship between remittances and economic growth can be impacted by factors such as financial development and policy changes.

However, the findings of this analysis were equivocal with regards to the statistical importance of the real exchange rate (REER) on the real gross domestic product (RGDP). This is in contrast to the conclusions drawn by Refs. [68,116]. A possible hindrance to Ethiopia's economic progress could arise from an upward movement in the exchange rate. Previous studies have underscored the need to adopt a suitable currency rate policy [66]. As previously stated, the maintenance of a stable exchange rate is crucial for promoting economic progress. The lack of empirical evidence establishing a causal relationship between the real effective exchange rate (REER) and economic growth suggests that other factors, such as domestic production capacity and trade policy, may have a more substantial impact on Ethiopia's economic growth. The position in question is supported by the scholarly works of [94]. Further work is necessary to address these inconsistencies and acquire a full grasp of the specific mechanisms involved.

Similarly, the performed research did not produce a statistically significant association between imports (IM) and real gross domestic product (RGDP). This observation is inconsistent with the findings reported by Refs. [37,90]. The lack of empirical evidence substantiating a direct link between imports and economic growth suggests that other variables, such as domestic production capabilities and trade policies, may play a moderating role in the impact of imports on economic development. Prior studies have presented a range of consequences for economic growth [61]. In their examination of the effects of imports on Ethiopia's economic growth [8,31], emphasize the academic importance of infrastructure and business legislation. Further investigation is required to have a deeper understanding of these complexities and align the research findings with existing scholarly studies.

Significant statistical associations were found between economic growth, as measured by real gross domestic product (RGDP), and two control variables: inflation (INF) and gross capital formation (GFCF). The coefficient of Gross Fixed Capital Formation (GFCF) has a statistically significant positive relationship (0.057***, p < 0.01), indicating the crucial role of investing in physical capital as a catalyst for economic growth. Previous scholarly research has established that investment plays a pivotal role in facilitating economic progress [65,66]. This remark underscores the need for implementing policies and adopting procedures that foster domestic investment. However, it is worth noting that [50,117] have highlighted the need for maintaining inflation at manageable levels in order to foster sustainable economic growth, as indicated by the statistically significant positive coefficient for inflation (0.027***, p < 0.01). The conclusions described above align with previous studies and underscore the significance of maintaining stable inflation rates and implementing efficient investment strategies to unlock Ethiopia's economic growth potential. Ethiopia's long-term economic development could potentially benefit from elevated levels of inflation. The findings of this discovery challenge the conclusions of prior research that has established a detrimental relationship between inflation and economic growth [75,118]. This statement emphasizes the importance of understanding inflation and economic progress within the specific context of Ethiopia. The current investigation has revealed a dearth of correlation between trade terms and long-term economic growth. Prior studies have demonstrated that the effects of trade on economic growth are not consistent [43]. This underscores the importance of considering additional variables that may interact with terms of trade, hence influencing economic growth.

To effectively address the identified deficiencies in the existing body of knowledge, forthcoming research endeavors should contemplate the investigation of multiple components. In order to gain a deeper understanding of the influence of foreign direct investment (FDI) and remittances on the economic growth of Ethiopia, it is crucial to examine the precise mechanisms via which these factors exert their effects. Moreover, a comprehensive analysis of the volatility of the real exchange rate and its relationship with economic growth will yield valuable insights into the importance of exchange rate policies and trade competitiveness. Ultimately, engaging in further investigation into the complexities pertaining to imports and their impact on economic growth would augment the overall understanding of Ethiopia's trading environment.

In conclusion, a favorable association has been observed between the coefficients of industrialization and long-term economic growth. The augmentation of the industrial sector's contribution to Ethiopia's gross domestic product (GDP) has the potential to bolster the country's long-term economic growth. According to Refs. [72,83], the findings of the literature show that industrialization is primarily what drives economic growth. This highlights the necessity for Ethiopia to embrace industrial development programs. In conclusion, this research contributes to the understanding of the complex relationship between the specified economic issues and the progress of Ethiopia's economic development. In order to effectively harness Ethiopia's economic potential in the context of intricate interconnections, it is imperative to employ tailored tactics and protocols. The study is in accordance with the theoretical perspective that an exchange rate that is deemed to be overvalued might impede the competitiveness of exports, while an exchange rate that is considered to be undervalued can facilitate growth driven by exports. This observation underscores the significance of incorporating exchange rate changes into the analysis of economic growth.

Additionally, previous studies by Ref. [109] provide empirical support for the claim that foreign direct investment (FDI) has a positive impact on economic growth. The theoretical framework recognizes that foreign direct investment (FDI) has the potential to stimulate economic growth through the facilitation of technology transfer, the enhancement of productivity, and the provision of access to new markets. This assertion is consistent with the theoretical framework that posits foreign direct investment (FDI) as having a favorable impact on economic development.

Furthermore, the research substantiates the hypothesis that remittances have a role in fostering economic expansion [12,20]. Research has demonstrated that remittances have the potential to enhance household income and foster investment, thereby contributing to a rise in overall economic growth. The theoretical framework substantiates this viewpoint by placing emphasis on the beneficial effects of remittances on household income and subsequent economic progress. Nevertheless, the findings of this study do not establish a statistically significant association between imports and economic growth. This outcome poses a challenge to the prevailing paradigm that posits a positive link between imports and economic growth. This implies that there may be other significant elements, such as domestic production capacity and trade policies that exert a greater influence on economic development. This study provides a significant addition to the current scholarly discourse by undertaking an examination of the impact of foreign direct investment (FDI), remittances, the real exchange rate, and imports on the economic growth of Ethiopia. The confirmation of theoretical frameworks that establish a correlation between foreign direct investment (FDI) and remittances, as well as economic growth, through empirical verification serves as supporting evidence for previous scholarly inquiries. However, the lack of empirical evidence supporting the concepts around the correlation between the real exchange rate and imports calls for further investigation. The importance of foreign direct investment (FDI) and remittances, along with effective management of inflation and investment strategies, is significant in promoting economic growth in Ethiopia.

### 4.9 Diagnostics and model stability test

The findings of the research study on the dynamic effects of FDI remittances, REER, and IM on LGDP in Ethiopia, using the ARDL technique, are shown in Table 11. The diagnostic tests were authorized to ascertain the model's validity and reliability, and the findings are given in a clear and succinct way. In conclusion, the results of the diagnostic tests indicate that the model is appropriately stated, since there is no indication of heteroskedasticity or autocorrelation. Furthermore, the test results demonstrate that the data has a normal distribution and there are no indications of any specification errors in the model [15,111]. The aforementioned results instill a sense of confidence in the model's capacity to effectively assess the dynamic effects of the factors of concern on the economic development of Ethiopia.

**Table 11:** Model specification and diagnostic tests.

Tests	Chi2/F-value	DF	Prob	Test type
Breusch-Pagan test	0.29	–	0.5915	Heteroskedasticity
White's test	38	37	0.4236	
Durbin–Watson	2.953	(2, 13)	0.0876	An autocorrelation
Breusch-Godfrey	3.499	(1, 14)	0.0824	
Shapiro-Wilk test	−1.445	–	0.92579	Normality
Skewness/Kurtosistests	0.36	–	0.8365	
Ramsey RESET	1.65	(3, 12)	0.2293	Specification

**Fig. 5:**
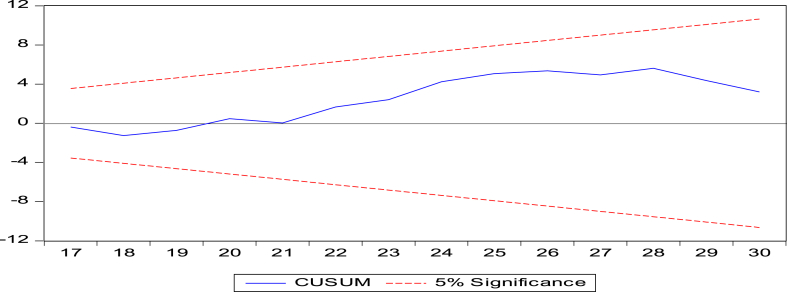
Cumulative sums.

**Fig. 6:**
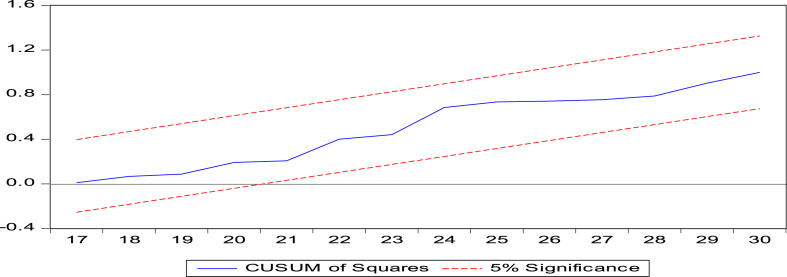
Cumulative sums of squares.

Fig. 5, Fig. 6 displays the CUSUM and CUSUMsq plots respectively, which indicate that neither of them exceeds the critical limits at a 5 % significance level. Therefore, we can conclude that the model is stable and can be used to analyze the variables of interest in this study.

### 4.10 Robustness analysis

Table 12 shows the robust analysis using FMOLS and CCR regression and their coefficients and t-statistics are shown in Table 12. Foreign direct investment (FDI) hurts economic development, whereas imports help. Also, the research recognizes the positive association between GFCF and economic growth. The FMOLS model shows a favorable link between remittances. This link is not statistically significant in the CCR model, but REER boosts economic growth. In contrast, inflation and terms of trade hurt economic growth. Industrialization is not correlated. These complex findings may help policymakers and researchers maximize Ethiopia's economic potential. This section analyzes FMOLS and CCR model data to assess the study results' robustness. These tests are vital to proving the links between economic conditions and Ethiopia's economic success.

**Table 12:** Robustness test results.

Variables	FMOLS	CCR
	Coef./t-stats.	Coef./t-stats.
FDI	−1.097***(-7.01)	−1.1165***(-4.87)
IM	0.450***(7.42)	0.4514***(5.35)
GFCF	0.199***(3.93)	0.204***(3.02)
REM	1.086***(2.62)	1.053*(1.66)
REER	0.0781***(8.82)	0.0782***(8.81)
IN	−0.108***(-5.76)	−0.113***(-3.30)
TOT	−0.0542***(-3.37)	−0.055***(-2.60)
INDU	0.993 (0.15)	1.305 (0.15)
Constant	−12.025***(-3.55)	−12.086***(-2.72)
N	40	40
R^2^	0.549	0.451

Note: The significance levels are denoted by asterisks, with ***, **, and * representing 1 %, 5 %, and 10 % significance, respectively**.**

Moreover, both models' FDI coefficients consistently show a negative effect on economic growth. These findings reflect the study's prior findings that increasing foreign direct investment (FDI) may slow Ethiopia's economic growth. This contrasts with prior studies showing that foreign direct investment (FDI) boosts Ethiopia's economy [31,52]. The relationship between FDI and economic development is complicated and varied. Additionally, both models show consistent import results, showing no statistically significant effect on long-term economic growth. This remark supports the study conclusion that imports affect economic growth, depending on several contextual factors. This is consistent with prior findings showing that imports affect economic growth differently [21].

Additionally, both models reveal that investing is always crucial due to their positive and statistically significant coefficient estimates for gross fixed capital formation (GFCF). This means capital generation is still vital to Ethiopia's economic success. Investment is vital to economic progress, according to studies (48). Both models' coefficient estimates for remittances support the studys' prior result that they have little influence on long-term economic growth. This contradicts past studies (such as [115]) that emphasized remittances' benefits to Ethiopia's economy. This implies considering other factors that affect this link.

Likewise, both models' real effective exchange rate (REER) factors consistently negatively affect long-term economic growth. This suggests that a stronger currency rate may hinder Ethiopia's economic growth. Previous studies emphasize the need for appropriate exchange rate laws [66]. The inflation coefficient estimates are consistent, demonstrating a strong positive relationship with long-term economic growth. Contrary to expectations, this unexpected result implies that high inflation rates may benefit Ethiopia's economic growth in the long term. This highlights the necessity of understanding inflation and economic progress in Ethiopia.

Moving to the TOT, the coefficient estimates in both models indicate no statistically significant influence on long-term economic growth, consistent with earlier studies. This supports a recent study that shows terms of trade affect economic growth differently [70], emphasizing the necessity to account for interacting factors. Finally, both models have positive industrialization coefficient estimates, but they are not statistically significant. Industrialization may boost Ethiopia's economic development, but the study models suggest it may not be a long-term driver. These robustness tests confirm the consistency and reliability of the earlier findings. The solid results across multiple models strengthen the links between these economic determinants and Ethiopia's economic success.

## 5 Conclusions

In summary, this research study offers significant contributions to the understanding of the economic factors that contribute to Ethiopia's achievements and presents noteworthy consequences for policymakers and scholars. This study presents a significant discovery that questions the prevailing beliefs pertaining to foreign direct investment (FDI). In contrast to prior research that placed emphasis on the favorable influence of foreign direct investment (FDI) on the economy of Ethiopia, the outcomes of this study consistently demonstrate a detrimental impact on the country's economic growth. This emphasizes the necessity of a comprehensive comprehension of the relationship between foreign direct investment (FDI) and economic development, hence pressing policymakers to meticulously assess the potential ramifications of augmenting FDI inflows.

. Moreover, the analysis of the study indicates that imports have no substantial impact on long-term economic growth. Nevertheless, it is imperative to take into account contextual variables when evaluating the influence of imports on economic development. This sophisticated perspective is consistent with prior scholarly investigations that have illustrated the complex and multidimensional nature of the correlation between imports and economic growth. Furthermore, this study highlights the pivotal significance of gross fixed capital formation (GFCF) in the economic prosperity of Ethiopia. Gross Fixed Capital Formation (GFCF) always has a positive and statistically significant coefficient in both regression models. This shows how important investment is for boosting economic growth. It is imperative for policymakers and stakeholders to accord priority to the development of capital in order to facilitate and maintain long-term economic growth.

Furthermore, the research outcomes provide insights into the significance of remittances, the real effective exchange rate (REER), inflation, terms of trade (TOT), and industrialization in the process of economic development in Ethiopia. The findings of this study demonstrate that, although there are variations observed across these parameters, the robustness tests provide evidence supporting the overall coherence and dependability of the initial results.

This study makes a valuable contribution to the current body of knowledge by conducting a thorough analysis of the economic factors that influence the economic performance of Ethiopia. Through the analysis of various regression models and the implementation of robustness tests, this study enhances the comprehension of the associations between these variables and the phenomenon of economic growth. The aforementioned discoveries possess consequences that extend beyond the geographical boundaries of Ethiopia, hence providing significant information of relevance to the scientific community.

In conclusion, this research provides significant contributions to understanding the economic factors that have contributed to Ethiopia's achievements. The ramifications of the findings are significant for policymakers and researchers, as they contribute to a more comprehensive comprehension of the intricate dynamics inside Ethiopia's economy. Through a comprehensive analysis of the intricate interconnections among foreign direct investment (FDI), imports, gross fixed capital formation (GFCF), remittances, the real effective exchange rate (REER), inflation, terms of trade (TOT), and industrialization, relevant parties can strategically determine courses of action that will effectively optimize Ethiopia's economic capabilities.

## 6 Policy recommendations

Based on the findings of this study, it is recommended that Ethiopian authorities and investors take measures to enhance the inflow of foreign direct investment (FDI) as a means to foster sustainable economic growth. These actions are expected to contribute to the economic development and resilience of the nation.

The study's findings highlight the significant role of foreign direct investment (FDI) in stimulating economic growth. To fully leverage this potential, policymakers must prioritize building and strengthening investor confidence. Achieving this objective requires optimizing administrative processes, enhancing legal accountability, and promoting ecologically responsible investments.

Furthermore, it is crucial to recognize the importance of remittances as a catalyst for immediate economic expansion. Authorities should actively explore strategies to redirect and utilize remittance inflows for fostering productive firms. Implementing financial literacy programs and tailored investment platforms that address individuals' specific requirements can facilitate informed financial decision-making, thereby contributing to economic growth.

Considering the intricate correlation between imports and economic growth, policymakers must adopt a comprehensive perspective. Trade policies should be carefully tailored to align with economic objectives. Strategic actions, such as implementing tariffs and providing domestic corporate incentives, can help strike a balance that promotes economic equilibrium.

In conclusion, policymakers need to thoroughly evaluate potential risks associated with remittances and imports, and devise strategies to effectively mitigate any adverse effects on overall economic growth. It is essential to emphasize the importance of further research to expand upon the existing findings and explore the impact of additional external factors, such as technology transfer and human capital development, on Ethiopia's economic progress.

## 7 Implications

The ramifications of the subject matter have substantial importance and justify the need for additional investigation. This study has substantial theoretical and practical implications. The incorporation of theory-driven research pertaining to external factors and their influence on the economic progress of Ethiopia is a valuable contribution to the existing academic discourse. The findings of the research offer valuable insights into the influence of foreign direct investment (FDI), remittances, and imports on economic growth. These lessons may offer benefits for other developing countries. The present study utilizes regression analysis as a robust methodology to investigate the external factors that exert an influence on economic growth. This methodology has the potential to be repeated in future research projects.

The current study utilized the DDT framework to investigate the economic growth of Ethiopia and the effects of foreign direct investment (FDI), remittances, the real exchange rate, and imports. Furthermore, the variables TOT, INDU, and INF have been duly considered. Prior studies have examined the factors that influence economic development using the DDT technique. However, there is a lack of research conducted in the eastern environment that investigates the correlation between these characteristics and predictions of economic progress. Hence, the inclusion of DDT within an eastern economic framework, coupled with the integration of control mechanisms, contributes to the enhancement of this research study from a theoretical standpoint. DDT has been essential in evaluating the effects of terms of trade (TOT), industrialization (INDU), and inflation (INF) on Ethiopia's economic progress. The aforementioned economic factors play a pivotal role in the progress of a nation's economy. The dearth of empirical evidence in this particular domain may be ascribed to previous scholarly inquiries [46,119] that failed to integrate these many economic elements into a complete framework to evaluate their combined influence on economic progress. Therefore, the inclusion of control variables and the application of the Difference-in-Differences Technique (DDT) in this study on economic dynamics and growth provide a significant contribution to the current academic discourse.

The findings of this study merit attention from policymakers and stakeholders with a vested interest in the economic landscape of Ethiopia. The study provides evidence that the influx of foreign direct investment (FDI) has a favorable effect on the economy of Ethiopia, potentially acting as a catalyst for the promotion of sustainable development. This study further argues that the influx of remittances and imports may have an adverse effect on short-term economic growth. Ethiopian policymakers and investors can employ this information to achieve long-term economic success.

The findings of this study have wide-ranging implications and hold significant importance for governmental bodies, investors, and researchers alike. This research presents a comprehensive analysis of the economic determinants that contribute to the accomplishments of Ethiopia, with the objective of offering pragmatic recommendations for fostering sustained economic growth in the country. These implications underscore the necessity of acquiring a complete understanding of key factors such as foreign direct investment (FDI), remittances, imports, and trade policy.

Furthermore, the study emphasizes the critical importance of cultivating investor confidence, enhancing administrative protocols, and promoting ecologically sustainable endeavors. These implications highlight the imperative for politicians to enact strategic steps that not only foster economic growth but also ensure environmental sustainability. By considering these consequences, stakeholders can make informed decisions that may enhance Ethiopia's economic prowess and promote long-term sustainable and inclusive progress. It is imperative to acknowledge the importance of these effects, as their implications extend beyond the borders of Ethiopia. This study offers valuable insights that can inform the economic strategy and decision-making processes of policymakers on a global scale. Investors possess the capacity to leverage these outcomes to discover potential investment opportunities and contribute to the economic progress of Ethiopia and similar economies.

Likewise, it is imperative to do further study in order to examine more comprehensively the impact of external factors, such as the transfer of technology and the growth of human capital, on the economic trajectory of Ethiopia. By augmenting the knowledge base within these particular fields, scholars possess the capacity to provide comprehensive perspectives and make significant contributions to the practice of evidence-based policymaking. In conclusion, the findings obtained from this research include extensive implications and hold significant significance. There exists a pressing requirement for a thorough understanding, strategic interventions, and more scholarly inquiry in order to facilitate the advancement of sustainable economic development in Ethiopia and other similar areas. Through the recognition and contemplation of these ramifications, political figures, financial stakeholders, and academic experts have the potential to collectively facilitate the realization of Ethiopia's economic potential and provide the groundwork for a prosperous trajectory.

## 8 Limitations

This research has major ramifications that require additional study. This regression analysis is a reliable way to examine external influences and Ethiopia's economic progress, but it has limits. The researcher analyzed just a few external variables using secondary data. Researchers might improve the current work by studying how technology transfer and human capital development affect Ethiopian economic progress. To further understand how external influences affect sector-specific economic development in Ethiopia, primary data sources should be used.

Institutional quality, political stability, good governance, and accountability may also affect economic drivers and development. Unfortunately, this study analysis ignored these aspects. Future studies should analyze these factors. Additionally, studying the threshold level of these growth drivers that would spur economic development in Ethiopia may provide significant insights. This might help policymakers and investors choose the best investment and policy intervention for Ethiopia's sustained economic development. This research has interesting insights, but its limitations must be acknowledged. The researcher mostly studies economic concerns, leaving political aspects untouched. Future studies should examine Ethiopia's complex political-economic relationship. This might provide a wealth of information on the nation's economic drivers. Finally, these policy proposals and limiting reflections provide a more balanced view of Ethiopia's economic path. Ethiopia can maintain economic growth by prioritizing strategic investments, remittances, and import management. By embracing political complexity, future research may uncover even more national growth and development potential.

## Consent to participate

Not Applicable.

## Consent to publish

Not Applicable.

## Disclosure of interest's statement

The author states that there are no competing interests to declare.

## Data availability statement

Data will be made available on request.

## Funding disclosure

The author did not receive any research funding for this paper.

## Transparency

The authors confirms that the manuscript is an honest, accurate, and transparent account of the study.

## CRediT authorship contribution statement

**Dereje Fedasa Hordofa:** Writing - review & editing, Writing - original draft, Validation, Software, Methodology, Data curation, Conceptualization.

## Declaration of competing interest

The authors declare that they have no known competing financial interests or personal relationships that could have appeared to influence the work reported in this paper.
